# AI Use for Mental Health Information and Professional Psychological Help-Seeking Intentions Among Saudi Women: A Cross-Sectional Study

**DOI:** 10.3390/bs16091642

**Published:** 2026-09-14

**Authors:** Nawal Alissa

**Affiliations:** Department of Community Health Sciences, College of Applied Medical Sciences, King Saud University, P.O. Box 10219, Riyadh 11433, Saudi Arabia; nalissa@ksu.edu.sa; Tel.: +966-11-8050987

**Keywords:** artificial intelligence, mental health, help-seeking behavior, psychological support, Saudi women, ChatGPT

## Abstract

Mental health concerns remain a major public health challenge, while artificial intelligence (AI)-based tools are increasingly used to obtain mental health-related information. However, little is known about the relationship between AI use and professional psychological help-seeking intentions, particularly among Saudi women. This cross-sectional study examined this association among 134 Saudi women aged 18 years and older recruited through social media platforms. An online questionnaire assessed AI use for mental health information, confidence in AI-provided information, perceived barriers to professional help-seeking, and professional psychological help-seeking intentions. Data were analyzed using descriptive statistics, Pearson correlation, and multiple regression. AI use was negatively correlated with professional psychological help-seeking intentions (r = −0.45, *p* < 0.001). Multiple regression analysis showed that greater AI use and higher perceived barriers were associated with lower professional psychological help-seeking intentions, whereas confidence in AI-provided information was not significantly associated with help-seeking intentions. These findings provide preliminary evidence of an association between AI use and professional psychological help-seeking intentions but do not establish causality. Given the cross-sectional design and smaller-than-planned sample, the findings should be interpreted cautiously. Larger longitudinal studies are needed to clarify this relationship and examine actual help-seeking behavior.

## 1. Introduction

Mental disorders remain one of the leading public health challenges worldwide, affecting hundreds of millions of people and contributing substantially to disability and reduced quality of life (World Health Organization, 2022). Conditions such as depression, anxiety, eating disorders, and bipolar disorder adversely affect psychological well-being, social functioning, and daily life. Although effective treatments are available, many individuals experiencing mental health problems remain untreated or undiagnosed because they do not seek professional care (Patel et al., 2018). Previous studies have identified several barriers to professional help-seeking, including stigma, preference for self-reliance, concerns about seeking psychological support, and difficulties accessing appropriate services (Corrigan, 2004; Vogel et al., 2007; Clement et al., 2015). These barriers may influence whether individuals seek professional care or turn to other sources of information and support.

In Saudi Arabia, mental health conditions have become an increasing public health concern. Depressive symptoms and psychological distress have been reported across different population groups, including primary care patients and university students, with women consistently demonstrating greater vulnerability than men (AlJaber, 2020; Elhessewi et al., 2021; Hakami, 2018). Studies have also identified elevated risks of eating disorders among Saudi female university students, highlighting the considerable mental health burden experienced by young women (El-Akabawy et al., 2022). Previous research has also identified stigma, preference for self-reliance, concerns about symptom severity, and limited awareness of available services as factors that may influence professional psychological help-seeking (Alissa, 2021; Alaqeel et al., 2023; Alhumaidan et al., 2024). These findings highlight the importance of understanding how Saudi women respond to mental health concerns and where they seek information and support.

At the same time, digital technologies are increasingly being used to obtain health and mental health information. Previous research has demonstrated the growing role of conversational agents and AI-based technologies in healthcare and mental health-related contexts (Laranjo et al., 2018; Abd-Alrazaq et al., 2019). AI-based tools can offer convenient access to information about symptoms, coping strategies, and treatment options. Their accessibility, privacy, and convenience may be particularly attractive to individuals who feel uncomfortable discussing mental health concerns with healthcare professionals or members of their social networks (Laranjo et al., 2018).

Recent evidence indicates that generative AI is becoming an increasingly common source of health information. A 2025 cross-sectional survey on online health information-seeking in the era of large language models found that 21.2% of participants had used LLM-based chatbots, including ChatGPT and Microsoft Copilot, to obtain health information. Among those who used these chatbots, 48.4% reported following the advice they received, while only 19.4% reported checking the information against other sources (Yun & Bickmore, 2025). Recent research has also begun to examine generative AI specifically for mental health-related needs. A 2025 mixed-methods study involving 270 regular ChatGPT users from 29 countries found that participants used ChatGPT for psychoeducation, understanding psychological symptoms, emotional support, companionship, and promoting well-being. Participants described accessibility, rapid responses, perceived quality of information, and freedom to express themselves as important benefits, while also raising concerns about information accuracy, limited personalization, and the lack of human interaction (Luo et al., 2025).

Although this emerging evidence suggests that generative AI is becoming part of how people seek and engage with health and mental health information, its relationship with professional psychological help-seeking intentions remains uncertain. From a health behavior perspective, obtaining information is an important component of the help-seeking process and may influence subsequent decisions about whether and where to seek care. AI-based conversational systems may therefore be associated with professional help-seeking intentions through several possible pathways. AI-generated information may increase mental health awareness, facilitate symptom recognition, reduce uncertainty about psychological symptoms, and provide information about available sources of professional support, potentially strengthening intentions to seek professional care. Conversely, if users perceive AI-generated information or emotional reassurance as sufficient to address their concerns, AI use may be associated with lower intentions to seek professional psychological support. Thus, AI use should not be assumed to promote or discourage professional help-seeking; rather, its relationship with help-seeking intentions should be examined empirically.

Current evidence regarding AI and mental health has focused primarily on the technical performance, feasibility, and clinical applications of conversational agents (Abd-Alrazaq et al., 2019; Laranjo et al., 2018). Comparatively little research has examined how AI use for mental health information relates to intentions to seek professional psychological help, particularly among women in Middle Eastern settings. This represents an important knowledge gap because women experience higher rates of common mental health conditions, including depression and anxiety, yet may encounter social, cultural, and psychological barriers to accessing professional mental health care (Seedat et al., 2009; World Health Organization, 2022).

From a behavioral perspective, examining this relationship is increasingly important as digital technologies become integrated into everyday health information-seeking behaviors. The present study conceptualizes AI use as a potential source of mental health information that may be associated with individuals’ perceptions and decisions regarding professional psychological care. Rather than assuming a causal direction, examining this association can provide preliminary evidence about whether greater use of AI for mental health information corresponds with stronger or weaker intentions to seek professional help.

Given the limited evidence in this area, particularly within Saudi Arabia, this exploratory study aimed to examine the association between AI use for mental health information and professional psychological help-seeking intentions among Saudi women. The study also examined whether perceived barriers to professional help-seeking and confidence in AI-provided information were associated with professional psychological help-seeking intentions.

Accordingly, the study addressed the following research questions:

RQ1: Is AI use for mental health information associated with professional psychological help-seeking intentions among Saudi women?

RQ2: Are perceived barriers to professional help-seeking and confidence in AI-provided information associated with professional psychological help-seeking intentions among Saudi women?

Based on these research questions, the following hypotheses were examined:

**H1.**  *Greater AI use for mental health information is associated with lower professional psychological help-seeking intentions.*

**H2.**  *Greater perceived barriers to professional help-seeking are associated with lower professional psychological help-seeking intentions.*

**H3.**  *Confidence in AI-provided information is associated with professional psychological help-seeking intentions.*

## 2. Methods

### 2.1. Research Design

This cross-sectional study examined the relationship between the use of artificial intelligence (AI) for mental health information and intentions to seek professional psychological help among Saudi women aged 18 years and older. Data were collected using an anonymous online questionnaire administered through Google Forms and distributed through WhatsApp, Telegram, and Instagram.

### 2.2. Study Population and Sample

The target population for this study was Saudi women aged 18 years or older residing in Saudi Arabia. Participants were eligible if they were Saudi women aged 18 years or older, resided in Saudi Arabia, were able to read Arabic, and provided informed consent. Women younger than 18 years and participants who submitted incomplete questionnaires were excluded. Participants were recruited using convenience sampling through WhatsApp, Telegram, and Instagram.

A minimum sample size of 385 participants was initially estimated using a 95% confidence level, a 5% margin of error, and an assumed response distribution of 50%. This initial estimate was based on a general survey-sampling approach for estimating a population proportion and was not specifically based on the Pearson correlation or multiple regression analyses. Data collection was conducted between October and November 2025. Recruitment took place within this predefined data-collection period and ended as planned in November 2025, despite the target sample size not being reached. A total of 161 responses were received, of which 27 were excluded because of incomplete questionnaires, resulting in a final analytical sample of 134 participants. No imputation was performed for missing data, and only complete questionnaires meeting the eligibility criteria were included in the analysis. No additional participants were excluded from the final analytical sample.

The smaller-than-planned sample size was acknowledged as a limitation of the study. Because the primary inferential analysis was multiple linear regression, a regression-focused sensitivity power analysis was subsequently conducted based on the final sample size to assess the detectable effect size and better align the power assessment with the analysis performed.

To maintain data quality, questionnaires were reviewed for completeness before analysis, and incomplete responses were excluded. Because the survey was anonymous and no personally identifying information was collected, duplicate responses could not be verified using personal identifiers.

### 2.3. Measures

Data were collected using a structured self-administered questionnaire consisting of validated instruments and study-specific items adapted from previous literature. The questionnaire included sections on demographic characteristics, psychological distress, AI use for mental health information, perceived barriers to professional help-seeking, confidence in AI-provided information, and professional psychological help-seeking intentions.

#### 2.3.1. Demographic Information

This section collected participants’ demographic characteristics, including age, educational level, occupation, and marital status. Participants were also asked to rate their overall mental health on a scale ranging from 1 (“Very poor”) to 10 (“Excellent”).

#### 2.3.2. Psychological Distress

Mental health status was measured using the Patient Health Questionnaire-4 (PHQ-4), a brief screening tool that assesses symptoms of anxiety and depression experienced during the past two weeks. The scale includes four items related to feelings of nervousness or anxiety, difficulty controlling worry, feeling down or hopeless, and loss of interest or pleasure in daily activities. Participants responded using a 4-point Likert scale ranging from 0 (“Not at all”) to 3 (“Nearly every day”), with a total score range of 0 to 12. Higher scores indicate greater psychological distress. PHQ-4 scores were categorized into four levels of psychological distress, with scores of 0–2 indicating normal distress levels, 3–5 indicating mild distress, 6–8 indicating moderate distress, and 9–12 indicating severe distress. The PHQ-4 has been shown to be a reliable and valid measure in community populations (Kroenke et al., 2009).

#### 2.3.3. AI Use for Mental Health Information

AI use for mental health information was assessed using a five-item measure developed by adapting concepts from previous research on digital health information-seeking behavior and the use of artificial intelligence in healthcare (Abd-Alrazaq et al., 2019; Laranjo et al., 2018). As no validated instrument specifically measuring AI use for mental health information among Saudi women was identified, the items were adapted for the objectives of the present study.

In this study, “AI use” refers to the frequency of using AI-based tools for mental health-related information and support. The measure focused on the purposes for which AI was used rather than on specific AI platforms or applications; therefore, individual AI tools were not analyzed separately. The scale assessed the frequency with which participants used AI tools to (1) search for mental health information, (2) better understand symptoms of stress, anxiety, or depression, (3) obtain coping strategies for emotional or psychological problems, (4) learn about available mental health treatments or management strategies, and (5) obtain emotional support or reassurance during periods of psychological distress.

Participants responded using a five-point Likert scale ranging from 1 (Never) to 5 (Always), with higher scores indicating more frequent use of AI for mental health-related purposes.

Because the scale was adapted for this study, content validity was evaluated by two experts in mental health and health education, who reviewed the items for relevance, clarity, and cultural appropriateness. The questionnaire was translated into Arabic using a forward-translation procedure and independently reviewed by two bilingual experts to ensure conceptual equivalence and linguistic accuracy. No quantitative content validity index was calculated; therefore, the content validity assessment was based on expert review rather than a numerical validity coefficient. The adapted five-item scale demonstrated good internal consistency in the present sample (Cronbach’s α = 0.84). Factor-analytic evaluation was not conducted; therefore, evidence regarding the dimensional structure and construct validity of the adapted scale is not available in the present study and should be established in future studies using larger samples and formal psychometric validation procedures.

#### 2.3.4. Barriers to Professional Help-Seeking

Barriers to seeking professional mental health support were assessed using items adapted from previous research on psychological help-seeking barriers and access to mental health services (Alissa, 2021; Corrigan, 2004; Vogel et al., 2007; Clement et al., 2015). Participants were asked, “What makes seeking professional mental health help difficult for you?” Response options included preferring to deal with problems alone, believing the problem was not serious enough, cost concerns, stigma or embarrassment, not knowing where to seek help, lack of nearby services, and distrust of mental health professionals. Participants were allowed to select all applicable barriers. For descriptive analyses, each barrier was summarized using frequencies and percentages. For the regression analysis, a total barriers score was calculated by summing the number of barriers selected by each participant, with higher scores indicating a greater number of perceived barriers to seeking professional mental health support.

#### 2.3.5. Confidence in AI-Provided Information

Confidence in AI-provided information was assessed using a single-item measure adapted from previous research on digital health technologies and AI acceptance in healthcare settings (Laranjo et al., 2018). Participants were asked, “How confident are you in the information provided by artificial intelligence?” Responses were recorded on a 5-point Likert scale ranging from 1 (Not confident at all) to 5 (Very confident), with higher scores indicating greater confidence in AI-generated mental health information.

#### 2.3.6. Intention to Seek Professional Psychological Help

Intention to seek professional psychological help was assessed using adapted items from the General Help-Seeking Questionnaire (GHSQ) (Wilson et al., 2005). Participants were asked, “For the following statements, please rate how likely you are to seek help from each of the following sources if you experienced a serious emotional problem.” Help sources included family members, close friends, mental health specialists, general practitioners, and artificial intelligence (AI). Responses were recorded on a 5-point Likert scale ranging from 1 (Not likely at all) to 5 (Very likely).

For descriptive analyses, each help source was summarized separately. For the regression analysis, responses for mental health specialists and general practitioners were combined because both represent formal healthcare-based sources of psychological support. The professional help-seeking intention score was calculated as the mean of these two items, with higher scores indicating greater intention to seek professional psychological help. The two-item measure demonstrated good internal consistency (Cronbach’s α = 0.81). A summary of the reliability and validity assessment of the study measures is presented in Table 1.

### 2.4. Data Analysis

Data were analyzed using IBM SPSS Statistics version 23 (IBM Corp., Armonk, NY, USA). Descriptive statistics were used to summarize participant characteristics and study variables. Categorical variables were summarized using frequencies and percentages, whereas continuous variables were summarized using means and standard deviations, as appropriate.

Internal consistency reliability was assessed using Cronbach’s alpha for applicable multi-item measures. Cronbach’s alpha was not applicable to the single-item Confidence in AI-Provided Information measure or the checklist of perceived barriers.

Pearson correlation analysis was used to examine the association between AI use for mental health information and professional psychological help-seeking intentions. Multiple linear regression analysis was conducted to examine the associations of AI use, perceived barriers to professional help-seeking, and confidence in AI-provided information with professional psychological help-seeking intentions. Statistical significance was set at *p* < 0.05, and effect estimates are reported with 95% confidence intervals where applicable.

Prior to analysis, assumptions for Pearson correlation and multiple linear regression were assessed. Linearity and homoscedasticity were examined using scatterplots and residual plots, multicollinearity was assessed using tolerance and variance inflation factor (VIF) values, and normality and independence of residuals were also examined. However, the detailed numerical diagnostic outputs were not retained and therefore could not be reported in the present manuscript.

A sensitivity power analysis was conducted using G*Power 3.1 to determine the minimum regression effect detectable with the final sample. With 134 participants, three predictors, α = 0.05, and 80% statistical power, the final sample was sufficient to detect an overall regression effect of approximately Cohen’s f^2^ = 0.08 or greater.

### 2.5. Ethical Consideration

Ethical approval was obtained from the Research Ethics Committee of King Saud University, Saudi Arabia (No: KSU-HE-25-1159).

## 3. Results

### 3.1. Participant Characteristics and Descriptive Findings

The study included 134 Saudi women. Participants’ demographic characteristics are presented in Table 2. Most participants were aged 18–30 years (59.7%), while 14.2% were older than 40 years. Regarding marital status, most participants were single (57.4%), followed by married (33.5%). Most participants had completed high school education (61.1%), and approximately one-third held a bachelor’s degree (34.3%). Only a small proportion reported elementary, secondary, or graduate-level education.

Regarding occupation, most participants were students (67.9%), followed by employed (23.1%) and unemployed (8.9%).

**Figure 1:**
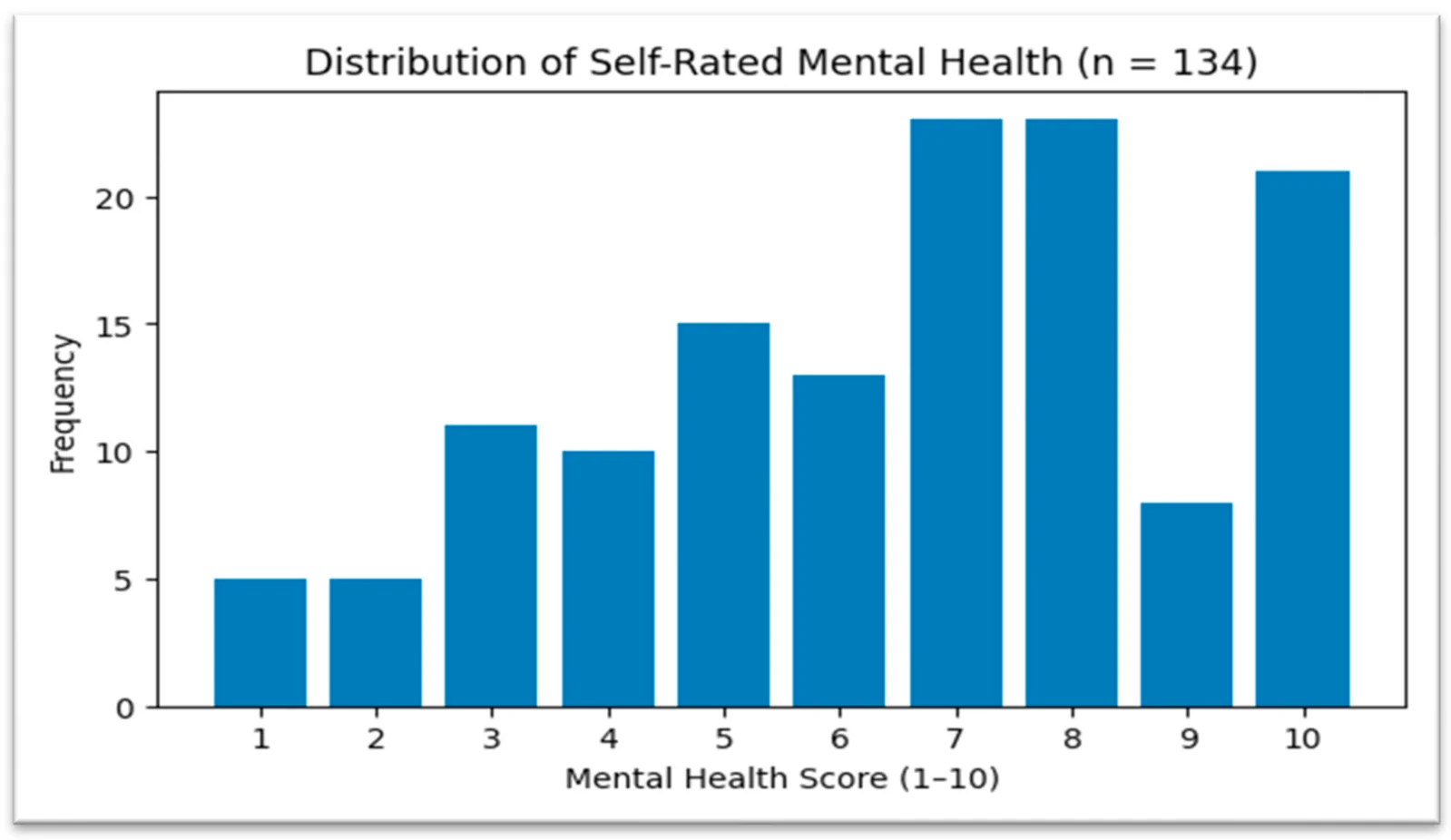
Self-Rated Mental Health Distribution.

More than half of the participants (55.6%) rated their overall mental health between 7 and 10 on the 10-point scale. Approximately one-fifth (21.0%) reported ratings of 5–6, whereas 23.4% reported ratings of 1–4 (Figure 1).

The mean PHQ-4 psychological distress score was 6.2 (possible range: 0–12), corresponding to the moderate psychological distress range. Regarding depressive symptoms (PHQ-2 items), more than 30% of participants reported experiencing little interest or pleasure in doing things or feeling down or hopeless either “more than half the days” or “nearly every day.” Anxiety symptoms (GAD-2 items) were more frequently reported, with 57.8% of participants reporting symptoms in these higher-frequency categories. Overall, anxiety symptoms were more commonly reported than depressive symptoms in this sample.

Participants reported relatively frequent use of AI tools for mental health-related purposes. The overall mean score on the five-item AI Use for Mental Health Information scale was 3.8 out of 5 (Table 3). Searching for mental health information had the highest mean score (M = 4.1, SD = 0.6), followed by using AI to understand symptoms of stress, anxiety, or depression (M = 3.9, SD = 0.7). Participants also reported using AI to seek coping strategies and learn about mental health treatments. Obtaining emotional support or reassurance during periods of psychological distress had the lowest mean score (M = 3.3, SD = 0.5).

**Figure 2:**
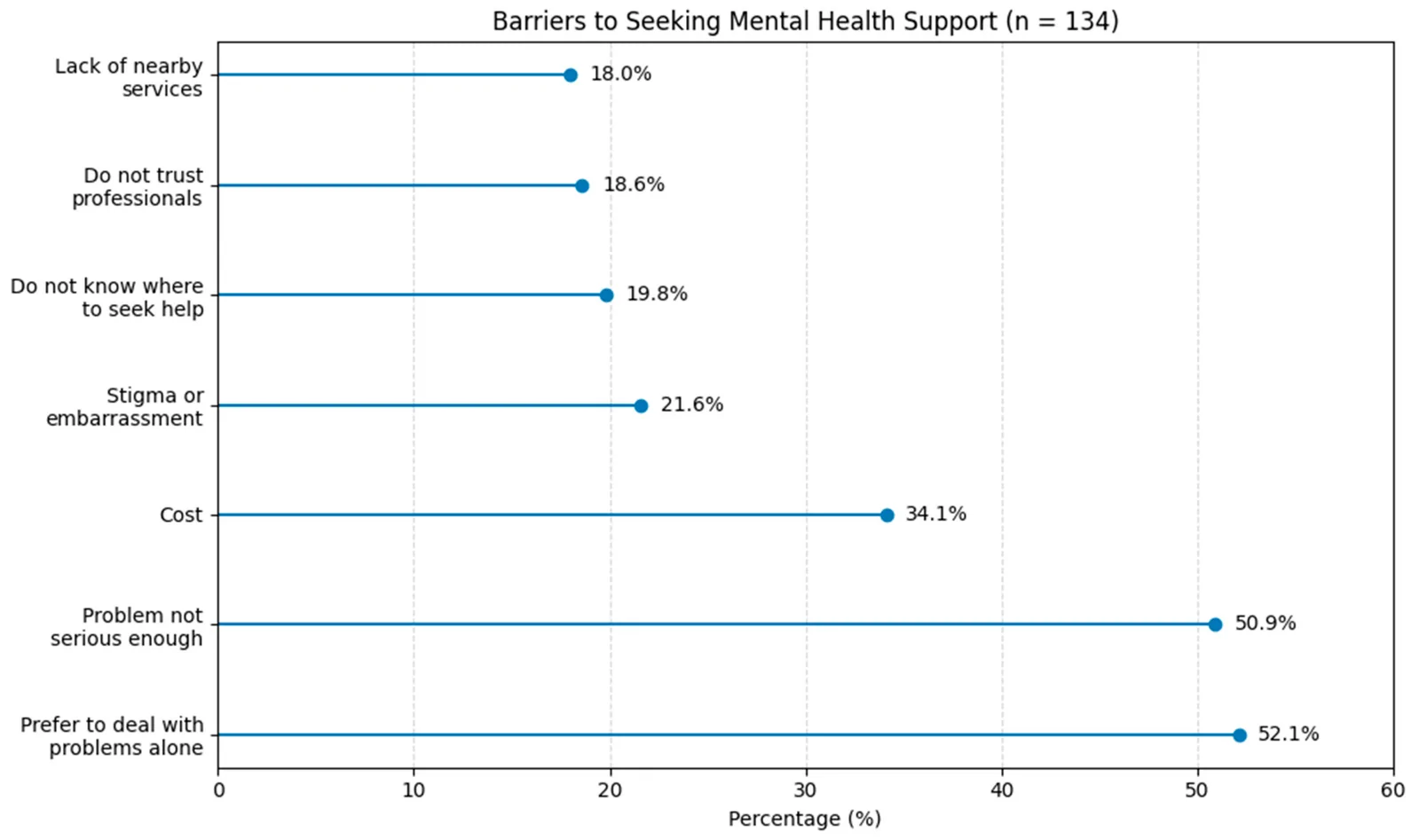
Barriers to Seeking Mental Health Support (N = 134).

Barriers to seeking professional help were summarized using frequencies and percentages based on participants’ multiple-response selections. The most frequently reported barrier was preferring to deal with problems alone (n = 87, 64.9%), followed by believing that the problem was not serious enough to require professional help (n = 85, 63.4%). Cost concerns were reported by 42.5% of participants (n = 57). Less frequently reported barriers included stigma or embarrassment (n = 36, 26.9%), not knowing where to seek help (n = 33, 24.6%), lack of trust in mental health professionals (n = 31, 23.1%), and lack of nearby services (n = 30, 22.4%) (Figure 2). Because participants could select more than one barrier, the percentages are not mutually exclusive and may sum to more than 100%.

Participants generally reported confidence in AI-generated mental health information. The most common response was “Somewhat confident” (58.1%), followed by “Very confident” (20.4%) and “Extremely confident” (6.6%). Lower confidence was less common, with 11.4% reporting low confidence and 3.6% reporting no confidence.

Participants’ intentions to seek help from different sources are presented in Table 4. Close friends were the most frequently endorsed source, with 34.3% of participants reporting that they were very likely to seek help from a close friend, followed by family members (31.3%). Among professional sources, 29.1% were somewhat likely and 23.9% were very likely to seek help from a mental health specialist. For general practitioners, 23.1% were somewhat likely and 17.2% were very likely to seek help. Regarding AI, 47.0% of participants were either somewhat likely or very likely to seek help from AI. The complete distribution of responses across all help sources is presented in Table 4.

### 3.2. Hypothesis Testing

#### 3.2.1. Association Between AI Use and Professional Psychological Help-Seeking Intentions (H1)

The association between AI use for mental health information and professional psychological help-seeking intentions was examined using Pearson correlation analysis. A statistically significant negative correlation was observed between AI use and professional psychological help-seeking intentions (r = −0.45, 95% CI [−0.58, −0.30], *p* < 0.001), indicating that greater AI use was associated with lower professional psychological help-seeking intentions. Therefore, H1 was supported (Table 5).

#### 3.2.2. Associations of AI Use, Perceived Barriers, and Confidence in AI-Provided Information with Professional Help-Seeking Intentions (H2 and H3)

The overall regression model was statistically significant, F(3, 130) = 12.45, *p* < 0.001, explaining 32% of the variance in professional psychological help-seeking intentions (R^2^ = 0.32; adjusted R^2^ = 0.30).

Greater AI use for mental health information was significantly associated with lower professional psychological help-seeking intentions (B = −0.42, 95% CI [−0.59, −0.25], β = −0.38, *p* < 0.001). Greater perceived barriers to professional help-seeking were also significantly associated with lower professional help-seeking intentions (B = −0.31, 95% CI [−0.52, −0.10], β = −0.27, *p* = 0.004), supporting H2. Confidence in AI-provided information was not significantly associated with professional help-seeking intentions (B = 0.08, 95% CI [−0.10, 0.26], β = 0.06, *p* = 0.381); therefore, H3 was not supported (Table 6).

## 4. Discussion

### 4.1. Association Between AI Use and Professional Help-Seeking

This exploratory cross-sectional study examined the association between AI use for mental health information and intentions to seek professional psychological help among Saudi women recruited through online social media platforms. Given the substantial shortfall from the planned sample size, these findings should be considered preliminary. Greater AI use was associated with lower intentions to seek professional psychological support; however, this association should be interpreted cautiously. Furthermore, given the cross-sectional design, the findings should be interpreted as associations rather than evidence of causal or temporal relationships.

The observed negative association between AI use and professional psychological help-seeking intentions may have several possible explanations. AI-based tools can provide immediate, private, and easily accessible mental health information and emotional reassurance, which may make them attractive to individuals experiencing barriers to professional services. This interpretation is consistent with previous research showing that digital mental health resources can provide accessible informational and emotional support without necessarily increasing engagement with formal mental health services (Minerva & Giubilini, 2023; Pretorius et al., 2019).

Reverse causation is also possible, whereby individuals who are already less inclined to seek professional psychological care may be more likely to use AI because of its accessibility and privacy. However, the present study did not assess whether AI was used instead of professional care. Therefore, the findings should not be interpreted as evidence that AI use replaces, delays, or reduces professional help-seeking. Further longitudinal research is needed to clarify the direction of this relationship.

The findings should also be interpreted in light of potential residual confounding and selection bias. The regression model did not include several potentially important factors associated with professional help-seeking, such as age, educational level, occupation, marital status, self-rated mental health, psychological distress, previous mental health service use, and perceived stigma. Some of these factors may also be associated with AI use and could therefore partly explain the observed association between AI use and professional help-seeking intentions. In addition, participants were recruited through convenience sampling using online social media platforms, which may have resulted in greater participation by women who were more familiar with digital technologies or interested in AI or mental health topics. These factors may limit the generalizability of the findings to the broader population of Saudi women. Future longitudinal studies with larger and more diverse samples are needed to clarify these relationships.

### 4.2. Preferred Sources of Support

The findings also showed that participants were more likely to seek emotional support from close friends and family members than from AI or healthcare professionals. This finding highlights the importance of interpersonal relationships as sources of support among the participants. Although participants generally reported moderate to high confidence in AI-provided mental health information, the findings do not suggest that AI was preferred over professional psychological care.

One possible explanation is that AI may be used for obtaining information, reassurance, or guidance, whereas professional mental health services provide individualized assessment and therapeutic support. However, the present study did not directly examine participants’ reasons for using AI or whether AI was intentionally used instead of professional psychological services. Therefore, this interpretation should be considered tentative and warrants further investigation.

The preference for family members and close friends may also reflect social and cultural influences on mental health help-seeking. Previous research has shown that help-seeking decisions can be influenced by stigma, attitudes toward professional psychological services, and other social and cultural factors (Alissa, 2021; Alaqeel et al., 2023; Alhumaidan et al., 2024). These findings highlight the importance of mental health services and digital initiatives that recognize the role of informal support while encouraging timely access to professional psychological care when appropriate.

### 4.3. Barriers to Professional Help-Seeking

The study identified several commonly reported barriers to professional psychological help-seeking. The most frequently reported barriers were preferring to deal with problems independently and believing that emotional difficulties were not serious enough to require professional support. These findings suggest that self-reliance and perceptions of symptom severity may be important factors associated with professional help-seeking among Saudi women recruited through online social media platforms.

Although stigma and embarrassment were reported less frequently, they may still influence decisions to seek professional psychological support. These findings are consistent with previous studies conducted in Saudi Arabia and other cultural settings, which have identified stigma, shame, and limited mental health awareness as barriers to accessing professional mental health services (Alaqeel et al., 2023; Alhumaidan et al., 2024; Alissa, 2021).

The accessibility and privacy of AI-based tools may offer an accessible source of mental health information for individuals experiencing barriers to professional support. However, the present study did not examine whether AI use reduced or overcame specific barriers to professional care. Therefore, its potential role in addressing help-seeking barriers remains unclear and requires further investigation.

### 4.4. Confidence in AI-Provided Information

Participants generally reported moderate to high confidence in AI-provided mental health information. However, confidence in AI-provided information was not significantly associated with professional help-seeking intentions when examined alongside AI use and perceived barriers in the regression model. This finding suggests that confidence in AI-provided information alone was not associated with participants’ intentions to seek professional psychological care. In contrast, the frequency of AI use showed a stronger association with professional help-seeking intentions.

This finding is broadly consistent with previous research reporting generally positive attitudes toward AI in healthcare (Syed et al., 2024). However, positive perceptions of AI do not necessarily indicate that individuals will change their intentions to seek professional psychological care. The present study adds preliminary evidence from a sample of Saudi women by examining confidence in AI-provided information alongside AI use and professional help-seeking intentions. Further research using larger and more diverse samples is needed to clarify the role of confidence in AI-provided information in mental health help-seeking.

### 4.5. Clinical and Public Health Implications

The findings highlight the importance of digital mental health literacy as AI becomes an increasingly accessible source of mental health information. Healthcare professionals can play an important role in helping individuals critically evaluate AI-generated information, recognize its limitations, and understand when professional psychological assessment may be needed. AI may provide convenient access to mental health information and initial guidance; however, it should complement rather than replace evidence-based psychological care. Public education initiatives that promote responsible AI use and digital mental health literacy may help individuals make informed decisions while reinforcing the importance of appropriate professional support.

### 4.6. Cultural Considerations

The Saudi cultural context should also be considered when interpreting these findings. Previous research has identified stigma, concerns surrounding mental health help-seeking, and other social and cultural factors as potential barriers to seeking professional psychological support in Saudi Arabia (Alissa, 2021; Alaqeel et al., 2023; Alhumaidan et al., 2024). In this context, the privacy and accessibility offered by AI-based tools may make them appealing as sources of mental health information. However, the present study did not directly examine whether cultural factors influenced participants’ decisions to use AI rather than professional services; therefore, this interpretation remains tentative.

These findings highlight the importance of considering cultural context when examining the role of AI in mental health support. AI may represent an accessible source of mental health information for some individuals; however, the present study does not establish whether AI encourages, delays, or replaces professional psychological care. Further research is needed to examine how AI use interacts with cultural and social factors and how AI may be appropriately incorporated into mental health services while supporting access to professional care.

### 4.7. Limitations

Several limitations should be considered when interpreting these findings. First, the study used convenience sampling, and participants were recruited through online social media platforms. This approach may have introduced selection bias, as women who use social media or are more familiar with digital technologies may have been more likely to participate. Consequently, the findings may not be generalizable to the broader population of Saudi women. Second, the achieved sample size (N = 134) was smaller than the estimated target sample of 385 participants. Although the sensitivity analysis indicated that the final sample could detect an overall regression effect of approximately Cohen’s f^2^ = 0.08 or greater, the smaller-than-planned sample may have reduced the precision and stability of the estimates, particularly for smaller effects.

Third, the study included only Saudi women; therefore, the findings may not be generalizable to men or other cultural or population groups. Fourth, all variables were measured using self-reported questionnaires and may therefore be subject to recall and social desirability biases. Fifth, the AI-use measure combined different purposes of AI use into a single overall measure and did not distinguish between specific AI platforms or applications. Therefore, potential differences in how particular types or purposes of AI use relate to professional help-seeking could not be examined. In addition, although content validity of the adapted AI-use measure was assessed through expert review and the scale demonstrated good internal consistency, no quantitative content validity index or factor-analytic evaluation was conducted. Further psychometric evaluation, including factor analysis in a larger independent sample, is therefore needed to establish its dimensional structure and construct validity.

Sixth, the regression model did not adjust for several potentially relevant demographic and psychological variables, including age, educational level, occupation, marital status, self-rated mental health, and psychological distress. Consequently, residual confounding cannot be ruled out, and the observed association may partly reflect the influence of unmeasured or uncontrolled factors. In addition, further statistical analyses and model diagnostics could not be conducted because the participant-level dataset is no longer available. This limited the ability to further examine the robustness of the observed association and the potential influence of confounding variables. These methodological limitations should therefore be considered when interpreting the magnitude and stability of the observed association.

Finally, the cross-sectional design precludes conclusions regarding causality or the temporal direction of the observed association. It cannot be determined whether greater use of AI for mental health information is associated with lower intentions to seek professional psychological help, whether women with lower help-seeking intentions are more likely to use AI, or whether both are influenced by other factors. Accordingly, the findings should be considered exploratory and interpreted with caution. Future studies using larger and more representative samples, validated measures, appropriate adjustment for potential confounders, and longitudinal designs are needed to confirm and extend these findings.

### 4.8. Implications for Practice and/or Policy

The findings suggest that AI is frequently used as a source of mental health information among Saudi women recruited through online social media platforms. Healthcare professionals can play an important role in helping individuals critically evaluate AI-generated information, recognize its limitations, and understand when professional psychological assessment may be needed. Promoting digital mental health literacy may support more informed use of AI while reinforcing the importance of appropriate professional care.

From a clinical perspective, AI may provide an accessible source of mental health information for some individuals, but it should complement rather than replace evidence-based psychological care. Given the observed association between greater AI use and lower professional help-seeking intentions, healthcare professionals may consider discussing the appropriate use and limitations of AI when providing mental health education and reinforcing the importance of professional assessment when psychological concerns persist or worsen. However, because the present study did not establish a causal relationship between AI use and help-seeking, these implications should be interpreted cautiously.

From a policy perspective, the findings support consideration of public education initiatives that promote responsible use of AI for mental health information. Evidence-informed guidance and digital mental health literacy initiatives may help individuals understand both the potential benefits and limitations of AI-generated information. Further research is needed before specific policies regarding the integration of AI into mental health services can be recommended.

## 5. Conclusions

This study found that Saudi women recruited through online social media platforms frequently used artificial intelligence (AI) tools for mental health information and reported generally moderate to high confidence in AI-provided mental health information. Greater AI use for mental health information was associated with lower intentions to seek professional psychological help. This finding should not be interpreted as evidence that AI use causes lower professional help-seeking intentions. Given the cross-sectional design, the direction of the relationship cannot be determined.

Participants were more likely to seek support from close friends and family members than from AI or healthcare professionals. In addition, self-reliance and perceiving emotional problems as not serious enough to require professional care were among the most commonly reported barriers to professional psychological help-seeking.

Given the exploratory nature of the study, the smaller-than-planned sample, and the use of convenience sampling through online social media platforms, these findings should be considered preliminary and may not be generalizable to the broader population of Saudi women. Further longitudinal studies using larger and more representative samples are needed to clarify the relationship between AI use and professional help-seeking and to better understand the role of AI as a source of mental health information.

## Figures and Tables

**Table 1:** Reliability and Validity Assessment of the Study Measures.

Measure	No. of Items	Cronbach’s α	Validity/Adaptation Assessment
AI Use for Mental Health Information	5	0.84	Content validity assessed by two experts in mental health and health education; Arabic version independently reviewed by two bilingual experts.
Professional Help-Seeking Intention	2	0.81	Adapted from the General Help-Seeking Questionnaire (GHSQ; Wilson et al., 2005).
Confidence in AI-Provided Information	1	N/A	Single-item measure adapted from previous research.
Perceived Barriers to Professional Help-Seeking	Checklist	N/A	Adapted from previously published measures and literature.

Note. Cronbach’s alpha was reported for applicable multi-item measures. Content validity of the AI-use measure was assessed through expert review; no quantitative content validity index or factor-analytic evaluation was conducted.

**Table 2:** Demographic Characteristics of Respondents (*N* = 134).

Characteristics	*n*	%
**Age (years)**		
18–30	80	59.7
31–40	35	26.1
Over 40	19	14.2
**Marital Status**		
Married	45	33.5
Single	77	57.4
Divorced	11	8.2
Widowed	1	0.7
**Educational Qualification**		
Elementary	2	1.4
Secondary	1	0.7
High School	82	61.1
Diploma	0	0.0
Bachelor’s	46	34.3
Graduate Studies	3	2.2
**Occupation**		
Students	91	67.9
Employed	31	23.1
Unemployed	12	8.9

**Table 3:** AI Use for Mental Health Information Scale (N = 134).

Items	M (SD)
Searching for mental health information	4.1 (0.6)
Understanding symptoms of stress, anxiety, or depression	3.9 (0.7)
Seeking coping strategies for emotional or psychological problems	3.7 (0.8)
Learning about mental health treatments or strategies	3.8 (0.4)
Obtaining emotional support or reassurance during periods of distress	3.3 (0.5)

**Note.** Responses were measured on a 5-point Likert scale ranging from 1 (Never) to 5 (Always), with higher scores indicating more frequent AI use for mental health-related purposes.

**Table 4:** Help-Seeking Intentions by Source (N = 134).

Source	Not Likely at All	Somewhat Unlikely	Neutral	Somewhat Likely	Very Likely	Total Responses
**Family Member**	18 (13.4%)	18 (13.4%)	22 (16.4%)	34 (25.4%)	42 (31.3%)	134
**Close Friend**	9 (6.7%)	15 (11.2%)	26 (19.4%)	38 (28.4%)	46 (34.3%)	134
**Specialist**	16 (11.9%)	19 (14.2%)	28 (20.9%)	39 (29.1%)	32 (23.9%)	134
**General Practitioner**	18 (13.4%)	28 (20.9%)	34 (25.4%)	31 (23.1%)	23 (17.2%)	134
**Artificial Intelligence (AI)**	20 (14.9%)	19 (14.2%)	32 (23.9%)	33 (24.6%)	30 (22.4%)	134

**Table 5:** Pearson Correlation Between AI Use for Mental Health Information and Intention to Seek Professional Psychological Help (N = 134).

Variables	r	95% CI	*p*-Value
**AI use for mental health information and intention to seek professional psychological help**	−0.45	[−0.58, −0.30]	<0.001

**Note.** CI = confidence interval.

**Table 6:** Multiple Linear Regression Analysis Examining Associations with Professional Help-Seeking Intentions (N = 134).

Predictor Variables	B	β	t	*p*-Value
**AI Use for Mental Health Information**	−0.42	−0.38	−4.82	<0.001
**Barriers to Professional Help-Seeking**	−0.31	−0.27	−2.95	0.004
**Confidence in AI-Provided Information**	0.08	0.06	0.88	0.381

Model Statistics: R^2^ = 0.32, Adjusted R^2^ = 0.30, F(3, 130) = 12.45, *p* < 0.001. **Note.** B = unstandardized regression coefficient; β = standardized regression coefficient.

## Data Availability

The participant-level data supporting the findings of this study are no longer available and therefore cannot be provided upon request.
